# Expression profiles and clinical significance of cystatin family genes in transitional cell carcinoma of the urinary bladder

**DOI:** 10.14440/bladder.2024.0057

**Published:** 2025-03-13

**Authors:** Haixia Xu, Wang Liu, Xiangxing Kuang, Jiang Zhao, Xiangwei Wang, Benyi Li

**Affiliations:** 1 Department of Medical Oncology, The First Affiliated Hospital of Shenzhen University, Shenzhen Second People’s Hospital, Shenzhen, Guandong 518035, China; 2 Department of Urology, School of Medicine, The University of Kansas Medical Center, Kansas 66160, United States of America; 3 Department of Urology, Guangzhou Hospital of Integrated Traditional and Western Medicine, Guangzhou, Guangdong 510803, China; 4 Department of Urology, School of Medicine, Guangdong Medical University, Zhanjiang, Guangdong 524001, China

**Keywords:** Bladder cancer, Cystatin family genes, Immune infiltration, Survival outcomes, Disease progression, DNA methylation

## Abstract

**Background:**

Cystatins, encoded by the *CST* gene family, are a superfamily of cysteine protease inhibitors involved in a wide array of biological functions, including immune modulation and antimicrobial defense. Cystatin proteins are implicated in tumor progression through multiple mechanisms. However, their roles in bladder cancer remain poorly understood.

**Objective:**

This study examined the expression profiles of the cystatin family genes and analyzed their correlation with clinicopathological parameters using the Cancer Genome Atlas Bladder Cancer RNA-seq dataset.

**Methods:**

The RNAseq dataset derived from the Cancer Genome Atlas project was utilized for the gene expression analysis on the XIANTAO online platform. The UALCAN online platform was used for the analysis of gene expression in molecular subgroups. The differences among various subgroups were statistically analyzed to define the significance.

**Results:**

Our results showed that *CST3, CST6, CST7, CSA*, and *CSTB* were predominantly expressed in bladder tissues. *CST6, CSTA*, and *CSTB* were upregulated, while *CST3* and *CST7* were downregulated in bladder cancer tissues. *CST1* and *CST2* were moderately expressed but significantly upregulated in malignant tissues. Specifically, malignant tissues could be effectively differentiated from benign tissues in terms of *CST1* expression, with an area under the curve value of 0.904. Upregulation of *CST2* was associated with multiple clinicopathological parameters, while downregulation of *CST3* was correlated with unfavorable outcomes in overall survival, disease-specific survival, and progression-free survival. Further analysis revealed that *CST7* expression bore an association with immune infiltration, suggesting that it plays a role in the modulation of immune cells.

**Conclusion:**

*CST* genes were distinctly expressed in bladder cancer with different clinical implications.

## 1. Introduction

Bladder cancer represents one of the most common cancers worldwide, with significant morbidity and mortality rates.^1^ Bladder cancer ranks among the top 10 most common cancers globally, with higher incidence rates in developed countries.^2^ It predominantly affects older adults, with the prevalence being higher in men than in women, and is strongly linked to specific environmental exposures and lifestyle factors.^3^ According to statistics, men are approximately 3 – 4 times more likely to develop bladder cancer than women.^3^ Most bladder cancers are diagnosed in individuals aged over 55, with the average age at diagnosis around 73.

The most significant risk factor for bladder cancer is smoking, which is culpable for roughly 50 – 65% of cases.^4^ Carcinogenic chemicals in tobacco accumulate in the bladder, damaging its lining urothelial cells. Workers in industries such as rubber, dye, leather, and chemical manufacturing are at an elevated risk due to prolonged exposure to carcinogenic compounds, particularly aromatic amines.^4^ Conditions that cause chronic bladder inflammation, such as recurrent urinary tract infections or long-term catheter use, also increase the risk of developing bladder cancer.^5^ In addition, family history and specific genetic mutations (e.g., mutations in *TP53* and *FGFR3*) are associated with increased bladder cancer risk.^6^

The most common type of bladder cancer is urothelial carcinoma (transitional cell carcinoma), which originates from the urothelial cells lining the bladder.^7^ Other less common types include squamous cell carcinoma and adenocarcinoma. The outcomes of bladder cancer are highly variable, depending on several prognostic factors, including tumor stage and grade, genetic characteristics, health status, and treatment options. The stage of bladder cancer (classified as non-muscle-invasive, muscle-invasive, or metastatic) is a critical prognostic factor. Early-stage (non-muscle-invasive) cancers are associated with a more favorable prognosis, whereas muscle-invasive or metastatic cancers are linked to poorer outcomes.^8^

Microscopically, tumor grade reflects the degree of abnormality of cancer cells. Low-grade tumors are generally less aggressive and have a better prognosis, while high-grade tumors are more likely to invade surrounding tissues and metastasize. Bladder cancer has a high recurrence rate, especially among those with non-muscle-invasive bladder cancer (NMIBC), where recurrence occurs in up to 70% of cases.^9^ Patients with recurrent bladder cancer tend to have a more unfavorable prognosis and may require protracted surveillance and repeated treatments. Progression from non-invasive to muscle-invasive disease significantly worsens prognosis, with survival rates dropping as the cancer invades deeper layers of the bladder wall or spreads to other parts of the body.^10^

Molecular profiling has identified several genetic alterations that may impact the prognosis of bladder cancer. For example, *FGFR3* mutations are commonly found in low-grade NMIBC and are associated with a better prognosis. *TP53* and *RB1* mutations are linked to high-grade, muscle-invasive bladder cancers, indicating a poorer prognosis. The expression levels of biomarkers, such as *Ki-67, HER2*, and *PD-L1*, have been found to be able to predict outcomes and guide treatment.^6^,^11^ However, novel biomarkers are still needed to predict outcomes and therapeutic responsiveness.

The cystatin family of genes encodes proteins known as cysteine protease inhibitors, which play vital parts in modulating protein turnover and maintaining cellular homeostasis.^12^ These genes have been gaining attention due to their involvement in various physiological and pathological processes, including inflammation, immunity, tumor progression, neurodegeneration, and cardiovascular diseases, among others.^12^ The cystatin family proteins inhibit cysteine proteases, particularly the cathepsin enzymes involved in lysosome protein breakdown. This inhibition helps regulate cellular processes that rely on controlled protein degradation, including tissue remodeling, apoptosis, and immune responses.^13^

The proteins encoded by these genes are categorized into three main types. Type 1 cystatins (Stefins) are found primarily within the cell. These proteins, such as cystatin A (CSTA) and cystatin B (CSTB), are small molecules lacking disulfide bonds and are expressed in various tissues. They act intracellularly, are often involved in cytosolic processes, and play essential roles in inflammation and cellular defense. Type 2 cystatins include 12 members, cystatin SN (CST1), cystatin SA (CST2), cystatin C (CST3), cystatin S (CST4), cystatin D (CST5), cystatin E/M (CST6), cystatin F (CST7), CTES5 (CST8), CTES7A (CST9), CTES7B (CST9L), CTES2 (CST11), and CSTL1.^14^ These proteins are secreted into body fluids, such as blood, saliva, and tears. Type 2 cystatins are known for their regulatory roles in extracellular environments, impacting tissue remodeling and immune cell migration.^14^ Type 3 cystatins (Kininogens) are large molecules and are mainly involved in blood clotting and inflammation. These cystatins are represented by proteins such as high molecular weight kininogen and low molecular weight kininogen. They participate in various processes, such as blood coagulation, inflammation, and regulation of the kallikrein–kinin system, which controls blood pressure and pain sensation.^15^

The cystatin genes have several important biological roles, principally on the strength of their ability to control the activity of proteases.^13^ Cystatin proteins inhibit lysosomal cysteine proteases (cathepsins B, H, L, and S), which are crucial for protein degradation within cells and tissues. Cystatins help regulate cell death, survival, and differentiation by controlling these proteases. Cystatin family proteins are essential in the modulation of immune responses.^16^ For instance, CST3 regulates the activity of proteases that degrade extracellular matrix (ECM) components, which affects immune cell migration and inflammation resolution. Moreover, cystatins defend cells against infections and maintain cellular stability. CSTB, for example, is linked to cellular defense against oxidative stress and inflammation.^17^ By controlling protease activity, cystatin proteins contribute to tissue repair and wound healing, thereby preventing excessive tissue breakdown while promoting appropriate cell migration and matrix deposition.^14^

Clinically, CST3 has been extensively studied for its roles in neurodegenerative disorders.^18^ Abnormal accumulation or mutations of *CST3* have been found to be associated with Alzheimer’s disease due to its ability to inhibit proteases involved in amyloid-β protein degradation, thus impacting amyloid plaque accumulation in the brain. CST3 is also clinically used as a biomarker of kidney function.^19^ It is a reliable indicator of glomerular filtration rate, offering an alternative to creatinine. Elevated CST3 levels are also associated with cardiovascular risk factors and are considered predictive of cardiovascular events, especially in patients with chronic renal disease.

The cystatin family’s role in regulating protease activity can influence tumor progression. Cystatin proteins can restrict tumor invasion and metastasis by inhibiting proteases that degrade the ECM. However, some tumors downregulate cystatin expression to promote tissue invasion, oncologically making these genes potential therapeutic targets or prognostic markers.^13^ Tumor progression tends to rely on the degradation of the ECM to facilitate cancer cell invasion and metastasis. Cysteine proteases, especially cathepsins B, L, and S, actively degrade ECM components, promoting cell migration and metastasis. Cystatins, particularly CST3, inhibit these proteases, potentially reducing ECM breakdown and thus limiting tumor invasion. In many cancers, cystatin levels are often decreased, leading to unregulated protease activity and facilitating cancer progression.^20^,^21^ Conversely, higher cystatin expression may impede metastasis by reducing protease activity, though some aggressive cancers may develop mechanisms to bypass this inhibition.^22^-^24^

Furthermore, cystatin family proteins influence the tumor microenvironment (TME), which consists of immune cells, fibroblasts, and ECM components. By regulating proteases in the TME, cystatins affect immune cell recruitment, angiogenesis, and fibroblast activation. This regulation is crucial, as a tumor-favoring TME can support tumor growth and immune evasion. For instance, CST3 modulates immune responses by affecting cytokine levels and the infiltration of immune cells into the tumor site.^25^ Dysregulation in cystatin expression can thus alter the TME, rendering it more permissive to tumor growth and immune suppression.^26^

Cystatin levels in blood and tissue samples have shown promise as diagnostic biomarkers. Elevated or decreased levels of cystatins, particularly CST3, are found in several cancer types.^27^,^28^ These levels can indicate protease imbalance, providing clues about tumor aggressiveness and proteolytic activity within the tumor. The diagnostic utility of cystatins is enhanced by their sensitivity to protease-related changes, even in early-stage cancers. Alterations in cystatin levels, detectable in blood or tissue biopsies, offer an early indication of cancerous processes. Combined with other markers, cystatin expression patterns can enhance diagnostic accuracy. Low cystatin level or loss of cystatin function is often associated with poor prognosis, as it can signal increased proteolytic activity, higher invasiveness, and metastatic potential.^29^ Studies have shown that in cancers, such as breast and prostate cancer, lower cystatin expression correlates with advanced stage, higher metastatic burden, and shorter survival.^30^ On the other hand, high levels of cystatins may be associated with less aggressive tumors and better outcomes as they counteract protease-driven processes that favor metastasis.^23^,^31^

In human bladder cancers, cystatin expression profiles have been relatively under-studied.^32^ Early studies showed that *CSTB* expression in tumors or urine serves as a reliable biomarker of tumor recurrence and disease progression.^33^ This notion was supported by a recent report indicating that *CSTB* mRNA expression levels are predictive of post-operative recurrence.^30^ In addition, *CST4* expression was found in an mRNA-miRNA bipartite network involved in bladder cancer progression.^34^ However, two reports did not agree regarding the utility of serum CST3 as a potential biomarker for bladder cancer diagnosis.^35^ In this study, we systemically analyzed the expression profiles of cystatin family genes in human bladder cancer tissues. Our results revealed that *CST1*/*CST2*/*CST6* genes were significantly upregulated, whereas *CST3*/*CTS7* genes were downregulated in bladder cancers. While *CST1* expression served as a potent diagnostic biomarker, *CST2* expression was significantly associated with disease progression. In addition, *CST3* expression was linked to favorable outcomes, whereas *CTS7* expression was highly correlated with tumor immune infiltration.

## 2. Materials and methods

### 2.1. Gene expression profiles and genetic alteration in prostate cancers and cell lines

The Cancer Genome Atlas Bladder Cancer (TCGA-BLCA) RNA-seq dataset was employed to examine the gene expression profiles at the mRNA level in bladder cancers, as described in our recent publications.^36^-^44^ Statistical analysis and visualization were conducted using the Xiantao online platform (https://www.xiantaozi.com/). We used two approaches: the case-matched pair comparison (19 cases) approach and the group cohort comparison (413 patient cases) with 19 benign samples. Gene expression levels were also compared among subgroups using the UALCAN platform (https://ualcan.path.uab.edu/). Gene expression data in bladder cell lines were downloaded from the Cancer Cell Line Encyclopedia dataset^45^,^46^ on the cBioPortal platform.

### 2.2. Assessment of patient survival

Utilizing the Kaplan–Meier curve method, we assessed three survival measures, including overall survival, disease-specific survival, and progression-free interval, on the Xiantao platform. The minimum *p*-value cut-off approach was used to stratify patients into low or high-expression groups.^47^ Data visualization was conducted on the Xiantao platform with the survminer package and ggplot2 package of the R package (version 4.2.1).

### 2.3. Correlation of gene expression with immune infiltrations

Gene expression at the mRNA level in the TCGA-BLCA dataset was used to analyze the correlation with immune infiltrations on the Xiantao platform using the R-Gene Set Variation Analysis package (version 1.46.0).^48^ Infiltrating immune cells were categorized into 24 types by employing the single-sample gene set enrichment analysis as described in a prior report.^49^ Correlations between gene expression levels and infiltration index were statistically assessed in terms of Spearman coefficients.

### 2.4. Statistical analysis

Gene expression levels were quantified using log_2_ (transcripts per million + 1) values and presented as the mean ± standard error of the mean. Analysis of variance was conducted for multiple group comparisons. Student *t*-test was performed to determine the significance of the differences between the two groups. The results were visualized using the R package (version 4.2.1) and GraphPad software (version 9.1.0).

## 3. Results

### 3.1. Cystatin family genes were aberrantly expressed in bladder cancers

**Figure 1:**
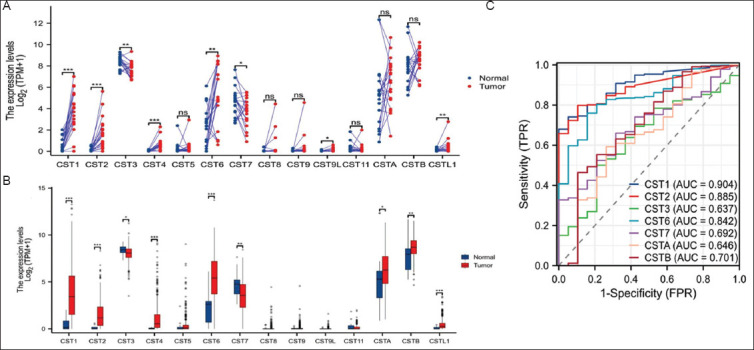
Dysregulation of cystatin family genes in prostate cancer. Case-matched pairwise approach (A) or group cohort comparison (B) were used to compare gene expression levels between benign and malignant tissues using the TCGA-BLCA dataset (log_2_ [value + 1). ROC analysis was conducted to determine the prediction potential in distinguishing normal and tumor tissues (C). Abbreviations: AUC: Area under the curve; FPR: False positive rate; TCGA-BLCA: The Cancer Genome Atlas Bladder Cancer; TPM: Transcripts per million; TPR: True positive rate; ROC: Receiver-operating characteristic curve.

We examined the expression profiles of the cystatin family genes in bladder cancers using the TCGA-BLCA RNA-seq dataset. Among the 14 protein-coding cystatin genes, *CST3* and *CSTB* were the predominant genes expressed in benign bladder tissues (Figure 1A). *CST1, CST6, CST7*, and *CSTA* genes showed moderate expression levels, whereas *CST2, CST4, CST5, CST8, CST9, CST9L, CST11*, and *CSTL1* were expressed at very low levels (Figure 1A and B). We then examined the differences in expression levels between benign and malignant bladder tissues using two approaches: case-matched pairwise and group cohort comparisons. In the pairwise comparison, *CST1, CST2, CST4, CST6*, and *CSTL1* genes were significantly upregulated, while *CST3* and *CST7* genes were significantly downregulated in malignant tissues (Figure 1A). With the group cohort comparison, the pairwise comparison revealed that all genes had the same trend of expression profiles (Figure 1B). In addition, *CSTA* and *CSTB* gene expressions were significantly higher in the malignant tissues than in their benign counterparts (Figure 1B). A receiver-operating characteristic curve analysis showed that *CST1* expression was most significant in distinguishing benign and malignant (area under the curve [AUC] = 0.904) tissues, followed by *CST2* and *CST6* (Figure 1C).

### 3.2. Aberrant expression of cystatin genes was associated with tumor progression in bladder cancers

**Figure 2:**
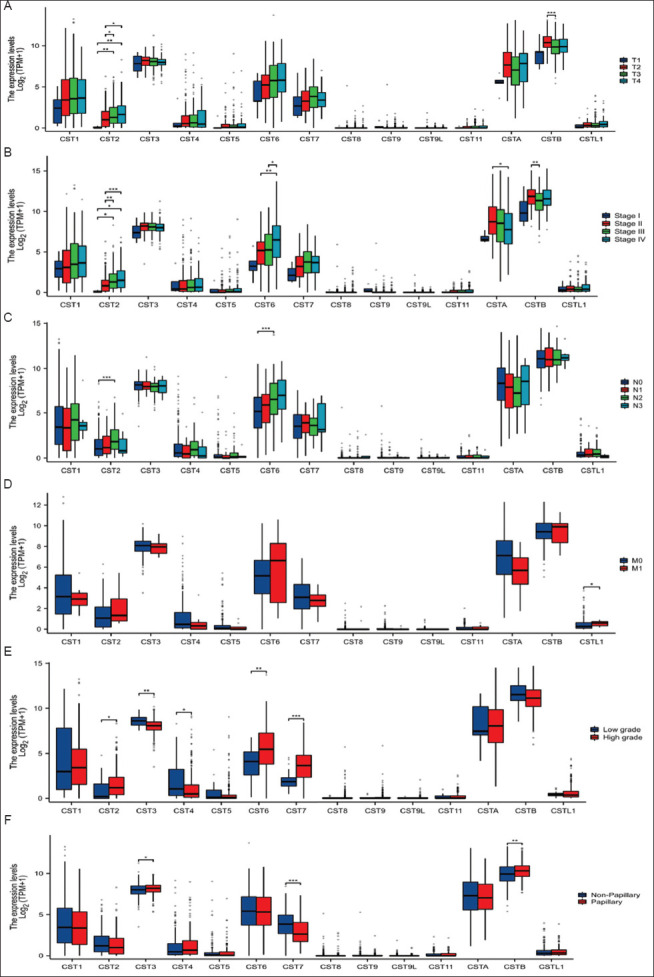
Cystatin gene expression in clinical subgroups. Pathological stage (A), clinical stage (B), lymph node invasion (C), distal metastasis (D), tumor grade (E), and tumor morphological types (F). The asterisks indicate a significant difference compared to the control group. Statistical significance determined at *p < 0.05; **p < 0.01, ***p < 0.001, and ****p < 0.0001. Note: T refers to Tumor in pathological stage, N refers to Lymph Node in lymph node invasion, and M refers to Metastasis in distal metastasis. Abbreviation: TPM: Transcripts per million.

We analyzed the association of gene expression levels with clinicopathological parameters to determine their clinical significance. *CST2* gene expression was the only one found to be highly correlated with increased pathological tumor stages (Figure 2A). *CST2* and *CST6* expressions significantly increased with late clinical stages, whereas the expressions of *CSTA* and *CSTB* were reduced in clinical stage III/IV compared to stage I (Figure 2B). Meanwhile, *CST2* and *CST6* expression levels significantly increased, which was associated with a higher incidence of lymph node invasion (Figure 2C). However, only *CSTL1* showed a marginally higher expression level in metastatic cases (Figure 2D). Compared to low-grade tumors, high-grade tumors exhibited significantly higher expression levels of *CST2, CST6*, and *CST7* but lower expression levels of *CST3* and *CST4* (Figure 2E). Compared to non-papillary tumors, papillary ones showed slightly higher expressions of *CST3* and *CSTB* but a significantly lower expression of *CST7* (Figure 2F).

### 3.3. Cystatin family genes were distinctly expressed in molecular subtypes of bladder cancers

**Figure 3:**
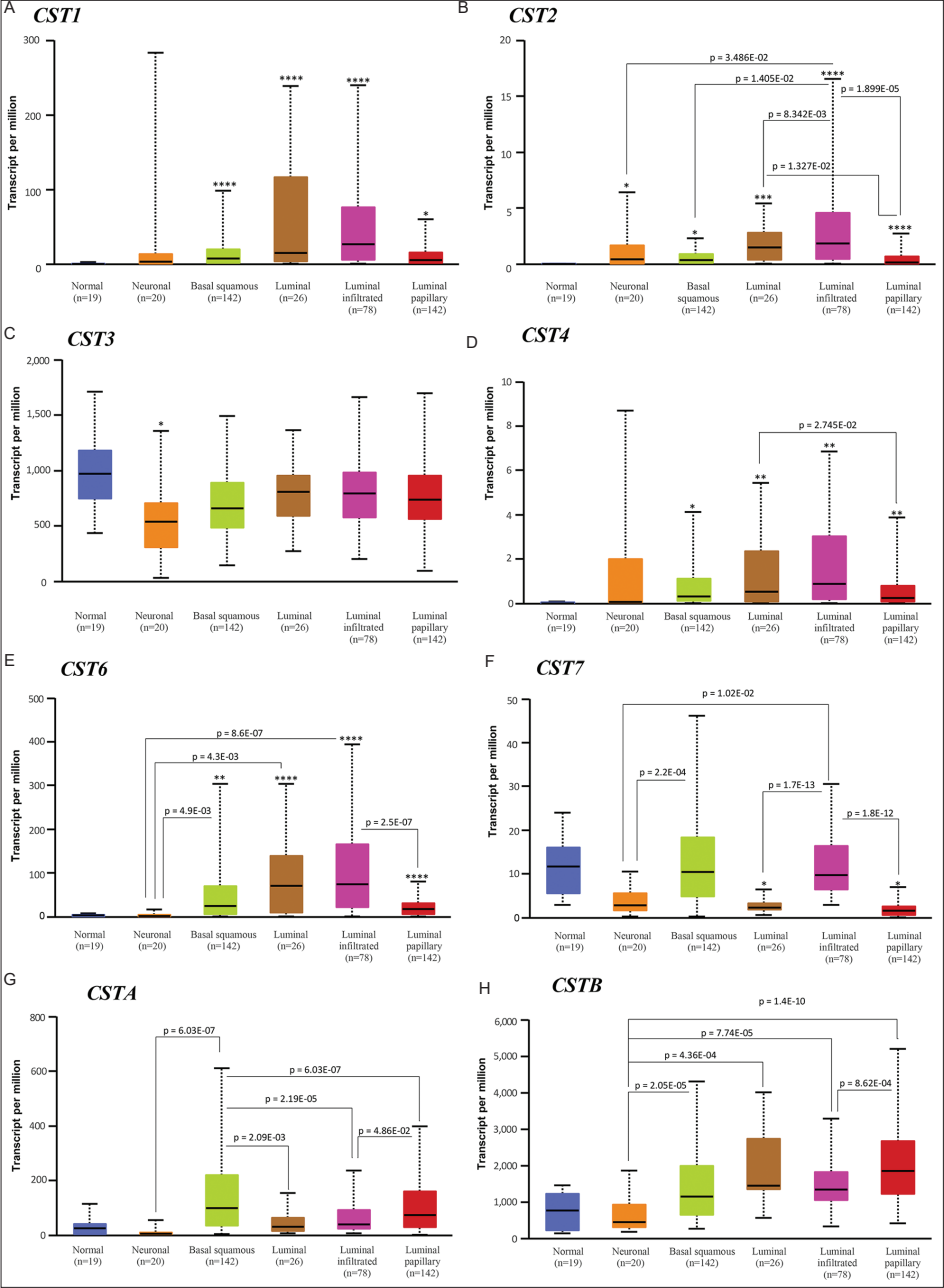
Cystatin gene family expression in molecular subgroups. Gene expression data for *CST1* (A), *CST2* (B), *CST3* (C), *CST4* (D), *CST6* (E), *CST7* (F), *CSTA* (G), and *CSTB* (H) were downloaded from the TCGA-BLCA dataset on the UALCAN platform. The asterisks indicate a significant difference compared to the benign group. Note: Student t-test; *p < 0.05; ***p < 0.001, and ****p < 0.0001. Abbreviation: TCGA-BLCA: The Cancer Genome Atlas Bladder Cancer.

We examined the expression profiles of cystatin genes in five different molecular subtypes.^50^ Although *CST1* expression was significantly higher in four of these subtypes, except the neuronal subtype, compared to benign tissues, there was no significant difference among these subtypes due to considerable variability (Figure 3A). Although the expression of *CST2* was very low in benign tissues, it was significantly upregulated in all five subtypes, with the highest expression found in the infiltrated luminal subtype, followed by the luminal subtype (Figure 3B). Interestingly, *CST3* expression was downregulated only in the neuronal subtype compared to benign tissues, with no significant differences revealed among the other subtypes (Figure 3C). *CST4* expression was lowest compared to other cystatin genes and was mainly found in the luminal subtype (Figure 3D). *CST6* was highly expressed in four subtypes, except the neuronal subtype, with the highest level being in the infiltrated luminal subtype (Figure 3E). In addition, *CST7* expression was downregulated in luminal and luminal-papillary subtypes and was significantly lower in neuronal subtype tumors than in basal squamous and infiltrated luminal subtypes (Figure 3F). However, *CSTA* and *CSTB* expression did not show statistically significant differences as compared to benign tissues (Figure 3G and H); the result is different from that of the group cohort comparison. Nonetheless, the *CSTA* gene was highly expressed in the basal squamous subtype, followed by papillary tumors (Figure 3G), whereas *CSTB* expression was primarily observed in luminal and papillary tumors (Figure 3H).

**Figure 4:**
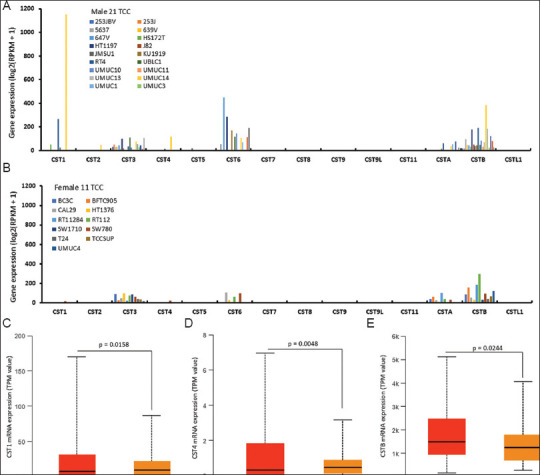
Cystatin gene expression in bladder cancer cell lines. Gene expression data were downloaded from the Cancer Cell Line Encyclopedia dataset on the cBioportal platform. Cell lines were divided based on the gender of the cell line origin: male (A) or female (B). Dedicated comparisons were shown for CST1 (C), CST4 (D), and CSTB (E). Abbreviations: n: Case number; TCC: Transitional cell carcinoma; TPM: Transcripts per million.

Moreover, we also examined cystatin gene expression profiles in human transitional cell carcinoma cell lines, including 21 lines from male and 11 from female patients. The significant isoforms were *CST3, CST6, CSTB*, and *CTSA* (Figure 4A and B). *CST1* was highly expressed in male patient-derived cell lines (Figure 4A).

### 3.4. *CST3* expression was associated with survival of bladder cancer patients

**Figure 5:**
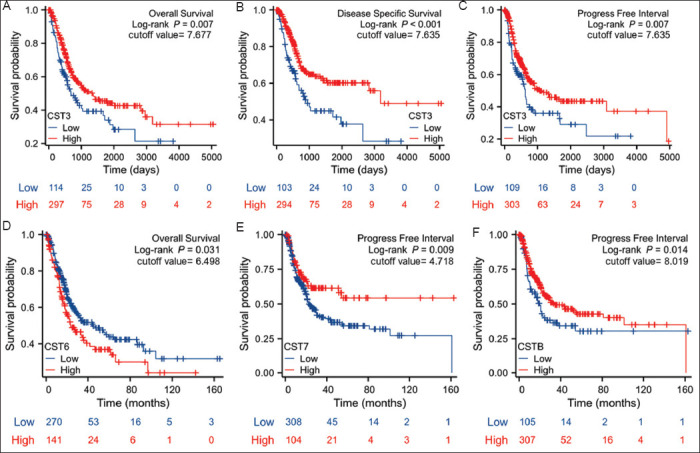
Kaplan–Meier survival outcome analysis. Gene expression data for the cystatin family genes were extracted from the TCGA-BLCA RNA-seq dataset. The Kaplan–Meier survival curve analysis was conducted with a minimum p-value approach on the Xiantao platform. A, CST3 overall survival; B, CST3 disease-specific survival; C, CST3 progression-free interval; D, CST6 overall survival; E, CST7 progression-free interval; F, CSTB progression-free interval.

We conducted a Kaplan–Meier survival curve analysis to examine the prognostic significance of aberrant expression of cystatin family genes. Our study revealed that only *CST3* expression was significantly associated with all three categories of patient outcomes, namely, overall survival (Figure 5A), disease-specific survival (Figure 5B), and progress-free interval (Figure 5C). These data suggest that *CST3* expression is a potential prognostic factor for bladder cancers.

In addition, higher *CST6* expression was significantly associated with poor overall survival, indicating a poor prognosis (Figure 5D). In contrast, *CST7* expression acted as a favorable factor for the progression-free interval (Figure 5E). Although *CSTB* was upregulated in malignant tissues in group cohort comparison, higher levels of *CSTB* expression were associated with a better outcome in progression-free intervals (Figure 5F). Therefore, its prognostic significance warrants further elucidation.

### 3.5. *CST7* expression was correlated with immune infiltration in bladder cancers

**Figure 6:**
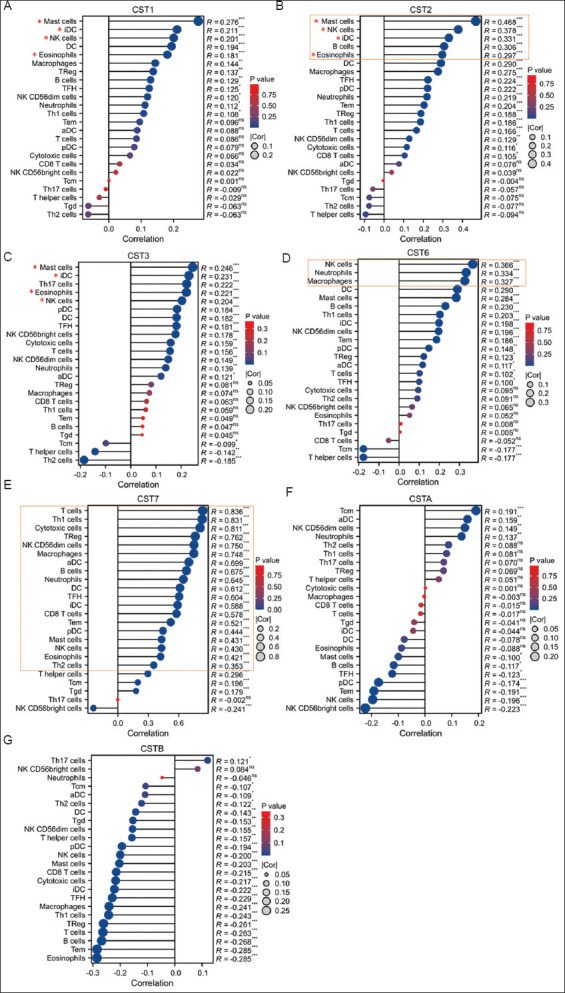
Correlation between cystatin family gene expression and immune infiltration. A, CST1; B, CST2; C, CST3; D, CST6; E, CST7; E, CSTA; G, CSTB. Gene expression at the mRNA levels (log2 [value + 1) was extracted from the TCGA-BLCA dataset, and the correlation analysis was conducted on the Xiantao platform. Note: ns refers to non-significant. Statistical significance determined at *p < 0.05; **p < 0.01, ***p < 0.001. Orange boxes denote strong correlations. Abbreviations: DC: Dendritic cells; NK: Natural killer; TCGA-BLCA: The Cancer Genome Atlas Bladder Cancer.

We assessed the correlation of the cystatin family genes with immune infiltration. Our analysis revealed that the expression levels of three cystatin genes, *CST1, CST2*, and *CST3*, were significantly correlated with four types of immune-infiltrating lymphocytes: mast cells, interstitial dendritic cells, natural killer (NK) cells, and eosinophils (Figure 6A-C), in which *CST2* expression showed the highest co-efficient levels (Figure 6B). In addition, *CST6* expression was significantly correlated with three infiltrating lymphocytes, NK cells, neutrophils, and macrophages, with co-efficient factors being more than 0.3 (Figure 6D). Interestingly, *CST7* expression was strongly correlated with virtually all immune-infiltrating lymphocytes, with a co-efficient factor higher than 0.3 (Figure 6E), indicating that *CST7* expression in bladder tissues may originate from the immune cells.^16^,^51^ In contrast, the expressions of *CSTA* and *CSTB* were only weakly correlated with one or two immune-infiltrating lymphocytes (Figure 6F and G).

## 4. Discussion

The cystatin family genes function as key players in cancer biology. They inhibit protease activity, limit tumor spread and invasiveness, and interact with immune and stromal components within the TME, influencing overall tumor behaviors. Given their involvement in these fundamental cancer events, cystatins hold significant potential as diagnostic and prognostic biomarkers as well as therapeutic targets in developing anti-cancer strategies. Their expression levels and functional status could provide valuable insights into cancer progression, offering tools for early diagnosis, monitoring, and assessment of treatment effects, ultimately contributing to more personalized and efficacious cancer management.

In this study, our results from benign bladder tissues and cancer cell lines showed that *CST3, CST6, CSTA*, and *CSTB* were the predominant isoforms out of the 14 family members expressed by bladder cancer cells. In bladder cancer tissues, *CST1, CST2, CST6, CSTA*, and *CSTB* were significantly upregulated, while *CST3* and *CST7* were downregulated compared to benign tissues. Among these altered genes, *CST1* could serve as a reliable diagnostic factor (AUC = 0.904), the finding being in line with previous reports on esophageal squamous cell carcinoma,^27^ endometrial cancer,^52^ and early-stage non-small cell lung cancer.^53^ In addition, our analysis revealed that *CST2* expression was associated with most clinicopathological parameters, indicating its possible role in tumor progression. *CST2* expression reportedly promoted tumor progression by activating the PI3K/AKT pathway in pancreatic cancer^54^ and was also associated with disease progression in colon cancer,^55^ prostate cancer,^56^ and gastric cancer.^57^

Cystatin C (*CST3*) is the most abundant extracellular inhibitor of cysteine proteases, with high concentrations of this protein found in biological fluids and practically all organs of the body, including the urinary bladder.^32^ In this study, we found that *CST3* expression was significantly associated with favorable patient outcomes, including overall survival, disease-free survival, and progression-free interval, consistent with previous findings on breast cancer,^58^ but not on non-small cell lung cancer.^59^

Urothelial bladder cancer is a heterogeneous epithelial malignancy with multiple subtypes, each exhibiting distinct molecular patterns and morphological features.^50^ Our results showed that *CST2, CST4*, and *CST6* were mainly expressed in infiltrated luminal tumors, whereas *CST1* and *CST3* showed no significant differences among subtypes. *CST7* expression was reduced in neuronal, luminal, and papillary tumors, while *CSTA* was highly expressed in basal squamous and papillary tumors. *CSTB* expression was higher in luminal and papillary tumors. This distinct expression pattern may mirror the molecular characteristics of cancer cells, warranting further investigation to gain mechanistic insights.

Cystatin F (*CST7*) expression is predominantly found in the bone marrow and spleen, suggesting a putative role in immune regulation.^51^ Although *CST7* expression has been reported in human cancer cells derived from malignant tumors,^16^ it has also been associated with patient outcomes.^60^ In this study, our analysis revealed that the *CST7* gene was not expressed in bladder cancer cell lines, and its expression in bladder tissues was lower than in benign tissues. Specifically, *CST7* expression was higher in high-grade, basal squamous nodular tumors. Interestingly, *CST7* expression was associated with most infiltrating lymphocytes in bladder cancers. These findings are coincident with recent reports on gastroesophageal cancers and cervical cancers,^61^,^62^ indicating that *CST7* plays a critical role in tumor immune regulation in bladder cancer.

## 5. Conclusion

Cystatin family genes were aberrantly expressed in bladder cancers, each having distinct profiles. Notably, *CST1* expression can serve to differentiate malignant tissues from benign tissues, suggesting that it may act as a potential diagnostic factor. In addition, higher *CST3* expression levels were associated with better survival outcomes, suggesting that it might be a favorable prognostic factor. *CST7* expression was correlated with most immune-infiltrating lymphocytes, highlighting its critical role in the immune environment.

## Data Availability

Data used in this study were generated from the TCGA Research Network (https://www.cancer.gov/tcga). Other data are available upon reasonable request to the corresponding author.
